# “Everything is from God but it is always better to get to the hospital on time”: A qualitative study with community members to identify factors that influence facility delivery in Gombe State, Nigeria

**DOI:** 10.1080/16549716.2020.1785735

**Published:** 2020-07-15

**Authors:** Zelee Hill, Pauline Scheelbeek, Joanna Schellenberg, Yashua Hamza

**Affiliations:** a Institute for Global Health, University College London, London, UK; b Department of Population Health, London School of Hygiene and Tropical Medicine, London, UK; c Disease Control, ChildCare and Wellness Clinics, Abuja, Nigeria; d Department of Disease Control, London School of Hygiene and Tropical Medicine, London, UK

**Keywords:** Facility delivery, Nigeria, qualitative

## Abstract

**Background:**

Nigeria has one of the highest maternal mortality rates in the world, but facility delivery levels are relatively low and stagnant. Few qualitative studies have explored this issue and most have focused on barriers to utilization, much can be learnt from women who already deliver in facilities.

**Objectives:**

We aimed to identify facilitators and barriers to facility delivery in Gombe State in North East Nigeria with a focus on women who have had a facility delivery.

**Methods:**

We conducted 24 narrative and in-depth interviews with mothers, and 16 focus-group-discussions with mothers, fathers, grandmothers and community health workers. Data were collected in Hausa, and transcribed and translated into English. Preliminary data analysis was conducted through team workshops, followed by systematic coding of the transcripts. Initial themes were identified a priori from the research questions and others emerged during coding.

**Results:**

A safe delivery was the main motivator for facility delivery, with facilities considered safe because of the presence of a trained health worker, the detection and management of problems, the availability of medicines and good hygiene. Those who delivered in a facility had a desire to be modern and rejected traditional practices. Decision-making power, social norms, accessibility, cost and perceived poor quality of care were reported as barriers. Community health workers, when they reached households, provided information on the benefits of facility delivery, stressed that times were changing, provided practical help such as arranging transport and, by accompanying families to the facility, brokered better quality of care and provided social support.

**Conclusion:**

This study highlights both the facilitators and barriers to facility delivery, and demonstrates the need for interventions to address a wide range of issues at multiple levels.

## Background

Maternal mortality rates are declining, but are still unacceptably high. Sub Saharan Africa has the highest burden of maternal deaths with a maternal mortality rate of 546 per 100,000 live births [1]. The 44% decline in maternal mortality observed in the region between 1990 and 2015, whilst laudable, is well below the target of a reduction of 75% [2]. Most maternal deaths are preventable, with facility delivery with a skilled attendant a key prevention strategy [3,4], yet in Sub Saharan Africa only 53% of deliveries are in a facility [5].

Nigeria has one of the highest maternal mortality rates in the world at 814 per 100,000 live births, and accounts for 19% of global maternal deaths [6]. Between 2003 and 2013 national facility delivery rates only increased from 33% to 36% [7], with the North Eastern Zone having facility delivery levels of 19% [8]. Despite this, little work has been done to understand the factors that influence the utilization of facilities for delivery. In particular qualitative studies, which can help understand the complexity of behaviours, are lacking. In a recent systematic review and qualitative synthesis of the barriers and enabling factors for facility delivery in low- and middle-income settings [9], only two Nigerian studies were identified, neither of which was classed as high quality [9]. A further three Nigerian studies were published subsequently [10,11,12]. These Nigerian studies suggest that in Nigeria decision-making power, accessibility, cost, social norms, cultural beliefs, fear of surgery, perceptions of quality of care, and a fear of disrespect and abuse by staff are barriers to facility delivery in Nigeria [10–14], which are similar to the findings of the systematic review in low- and middle-income countries [9]. More studies on factors that affect facility utilization in Nigeria have been called for [15], and studies from the Northern States are particularly lacking.

In this paper we report the findings of a qualitative study in Gombe State in North East Nigeria to identify facilitating factors and barriers to facility delivery, with a focus on learning from those who delivered in a facility. Understanding the behaviours of those who have delivered in a facility provides important lessons on the drivers of positive behaviours in relation to themselves [16] and also to women who do not yet deliver in facilities. In particular, respondents are often better at explaining other people’s behaviour than their own, and reflecting on the behavioural drivers of others reduces social desirability bias [17,18].

### Methods

Data were collected in 2015 as part of a larger study on how Community Health Workers (CHWs) influence maternal and newborn care in Ethiopia and Nigeria. In Nigeria data were collected from four Local Government Areas (LGAs), which correspond to districts, in Gombe State.

### Study setting

Gombe is multi-ethnic, predominantly rural and agrarian, 62% of females have no education, and the median age of marriage for women is 16 years [8,19]. The estimated 3.3 million population are served by 615 health facilities [20,21], with 28% of women delivering in a health facility, and 58% of pregnant women having an ante-natal care contact with a skilled provider. Almost all (98%) facility deliveries are in public facilities [8].

Volunteer CHWs, supported by the Society for Family Health (SFH), were active in the State at the time of data collection and made antenatal and postnatal home visits to improve service utilization and maternal and newborn care behaviours. They underwent 5–6 days of training and, although they are volunteers, they received incentives for accompanying women to facilities for delivery. SFH is one of Nigeria’s largest NGOs and has a wide range of programmes throughout the country. In Gombe, supported by the Bill and Melinda Gates Foundation, they trained and supported 1450 CHWs and ran an emergency transport scheme and a call centre focusing on maternal and newborn health. In urban areas CHWs were recruited through the Federation of Muslim Women’s Associations in Nigeria (FOMWAN) and in rural areas they were Traditional Birth Attendants. Coverage of home visits was low with only 29% of eligible women reporting that they had ever received a home visit [22].

Health system challenges faced throughout Nigeria, such as inadequate funding, irregular payments, lack of basic resources, insufficient and inequitably distributed health workers, strikes, and rapid population growth [23–26], were exacerbated in the North East by insurgent activities which reduced the numbers of health workers and resulted in large numbers of internally displaced populations [27,28].

## Site selection

We collected data from two LGAs in Gombe State that were considered to be typical of the area in terms of having no unusual characteristics such as industry or cash crops. Reflecting the religious diversity, we purposively selected one Muslim and one Christian LGA. In addition, an inclusion criterion was that the LGA needed to have a reasonably functioning CHW system so, with guidance from SFH, we selected LGAs that had CHWs in place whom SFH reported were actively making home visits. Insurgents were active during data collection and safety was a key consideration in site selection. Thus, for selection, LGAs had to be within a few hours’ drive of the State capital so the data collection team could return to the capital before dusk. Due to the proximity to the capital, study respondents are likely to have had better access to services than in the more remote areas of Gombe, including private health services. Within each LGA we selected an urban area (the LGA headquarters) and an accessible rural village.

### Data collection methods

We explored lived experiences of delivery through narrative interviews [29], during which respondents told the story of their last delivery. In-depth interviews (IDIs) were used to gain a perspective on community perceptions, attitudes and beliefs towards facility delivery by asking respondents what was commonly done in their community [30]. Focus group discussions (FGDs) focused on social norms and on topics that we felt would benefit from being discussed, and they utilized techniques to encourage interaction and discussion such as pile sorts [31]. Data were collected in each study site in turn, beginning with narratives and IDIs and then moving to FGDs. There were five topic guides in total reflecting the different data collection methods and respondent groups.

Data were collected in Hausa by four interviewers who used pre-tested semi-structured guides, the content of which was informed by the literature on factors that influence facility delivery [9] and the COM-B (‘capability’, ‘opportunity’, ‘motivation’ and ‘behaviour’) behaviour change model [32]. The pre-test was conducted after training in a location close to the training venue. Five interviews were conducted with respondents selected to be similar to those expected in the study area. After the pilot, the guides were revised in terms of their length and question formulation.

The interviewers, all graduates, had varying experience in qualitative data collection (between 2 and 17 years), and were supervised by a post-doctorate Nigerian experienced in qualitative research. Data were collected until saturation was reached, that is when data began to repeat [33]. This was determined through an iterative process of reviewing transcripts and conducting preliminary data analysis during data collection with saturation reached on all major themes. The sample size and the interview content related to facility delivery for each respondent group are shown in Table 1.

**Table 1.** Data collection method, sample size.

Method	Sample size	Examples of topic guide content
Narrative interviews with mothers of children under 3 months of age	12	Labour and delivery narrative Contact with health worker and CHW in pregnancy and at delivery Information received on where to deliver, reaction to the information, and influence of the information
In-depth interviews with mothers of children under 6 months of age	12	Perceptions of where most women deliver and what/who influences the decision Community views of those who deliver at home and those who deliver in a facility Most significant maternal and newborn health changes in last 2 years, and reasons for the change
FGD with mothers of children under 12 months of age	4	Pile sort of behaviours, such as facility delivery, early breastfeeding and delayed bathing, into those practised/not practised in the community Root causes of why facility delivery does not always happen Most significant maternal and newborn health changes in the last 2 years, and reasons for the change
FGD with grandmothers of grandchildren under 12 months of age	4	Reaction to a picture of a facility delivery Grandmothers’ role in delivery and in decision-making Reaction to statements about grandmothers supporting traditional practices, and about families not liking CHW advice on facility delivery Most significant maternal and newborn health changes in the last 2 years and reasons for the change
FGDs with fathers of children under 12 months of age	4	Reaction to a picture of a facility delivery Father’s role in delivery and in decision-making deciding place of delivery Reaction to statements about grandmothers supporting traditional practices, and about mothers/fathers making decisions about place of delivery alone Most significant maternal and newborn health changes in the last 2 years and reasons for the change
FGD with CHWs	4	Pile sort of behaviours, such as facility delivery, early breastfeeding and delayed bathing, into those practised/not practised in the community Root cause of why facility delivery does not always happen Most significant maternal and newborn health changes in the last 2 years and reasons for the change Successes and challenges that CHWs face in encouraging facility delivery

### Respondents

To capture a range of viewpoints data were collected from recent mothers, grandmothers, fathers, and CHWs. Community respondents were eligible if they were aged >18 years, had a young child/grandchild (less than 6 months of age for the mothers and less than 12 months of age for fathers and grandmothers) and their family had received at least one visit by a CHW. We used maximum diversity sampling principles to reflect the LGAs diversity in age, religion, and education. Community respondents were identified through the leaders of women’s groups, through CHWs, at places of worship and through snowball sampling. CHWs were identified with the assistance of SFH and through snowball sampling; there were no inclusion or exclusion criteria for the CHWs. Interviewers approached potential respondents in their home, explained the study and obtained written informed consent; no one refused to be interviewed.

Interviews were conducted in respondents’ houses and FGDs took place with 4–6 respondents in neutral locations such as schools. Interviews and FGDs lasted from 45 minutes to 2.5 hours, were audio-recorded and transcribed verbatim into English by the interviewers as soon as possible. Interviewers were trained and supervised to transcribe and translate with conceptual, and where possible semantic, equivalence and to capture the features of speech [34]. Ethical approval was granted by the National Health Research Ethics Committee of Nigeria (NHREC), the Gombe State Government and the London School of Hygiene and Tropical Medicine (reference 6088).

### Data analysis

We took a thematic approach to analysis [35]. Interviewers met regularly during fieldwork to discuss emerging themes and to receive feedback on their transcripts from the senior researchers. Team analysis workshops were held in the middle and at the end of data collection which, together with the content of the topic guides, formed the base of a deductive coding template. Once all the data were collected, transcripts were read several times to examine the data as a whole and identify a first set of inductive codes and themes. Each transcript was then inductively coded within the deductive themes by one of three senior researchers (ZH, YH, PS). The coding focused on the underlying meaning of the text and differences and similarities to other interviews. Conceptually similar codes were put into larger themes; and themes and codes were then modified by identifying patterns, links and contradictions within the data. Data credibility was checked by coders comparing and discussing their coding and by comparing and contrasting findings between respondent groups and data collection methods. This triangulation was done by analysing each respondent group and data collection method separately and comparing themes.

Table 2 shows the characteristics of the narrative and IDI sample. Half of the respondents were aged 25–34 years of age and had no education. Respondents had a range of parities, religion and place of residence, 20/24 women had delivered in a facility. The high number of women who delivered in a facility is linked to working in more accessible areas, and with women who had received at least one visit by a CHW.

**Table 2.** Sample characteristics (narrative and mother IDIs).

Characteristic	Frequency
**Age** ≤24 25–34 ≥35	8 12 4
**Education** None Primary Secondary and above	12 4 8
**Religion** Islamic Christian	15 9
**Parity** 1 2–4 ≥5	3 11 10
**Place of last delivery** Home Facility	4 20
**Residence** Urban Rural	12 12

The FGD mothers were varied in age (range 20–40 years of age), parity (range 1–8 children), education (two FGDs were with women who had not attended school and two with women who had attended), ethnicity and residence (two FGDs were in urban and two were in rural areas). The FGD fathers were older (range 30–61 years of age) and more educated than the mothers (most fathers had a least secondary level education). Most mother and father respondents (38/44) had the recent birth in a facility. The FGD grandmothers were less educated with almost all having no schooling.

## Results

**Figure 1.**
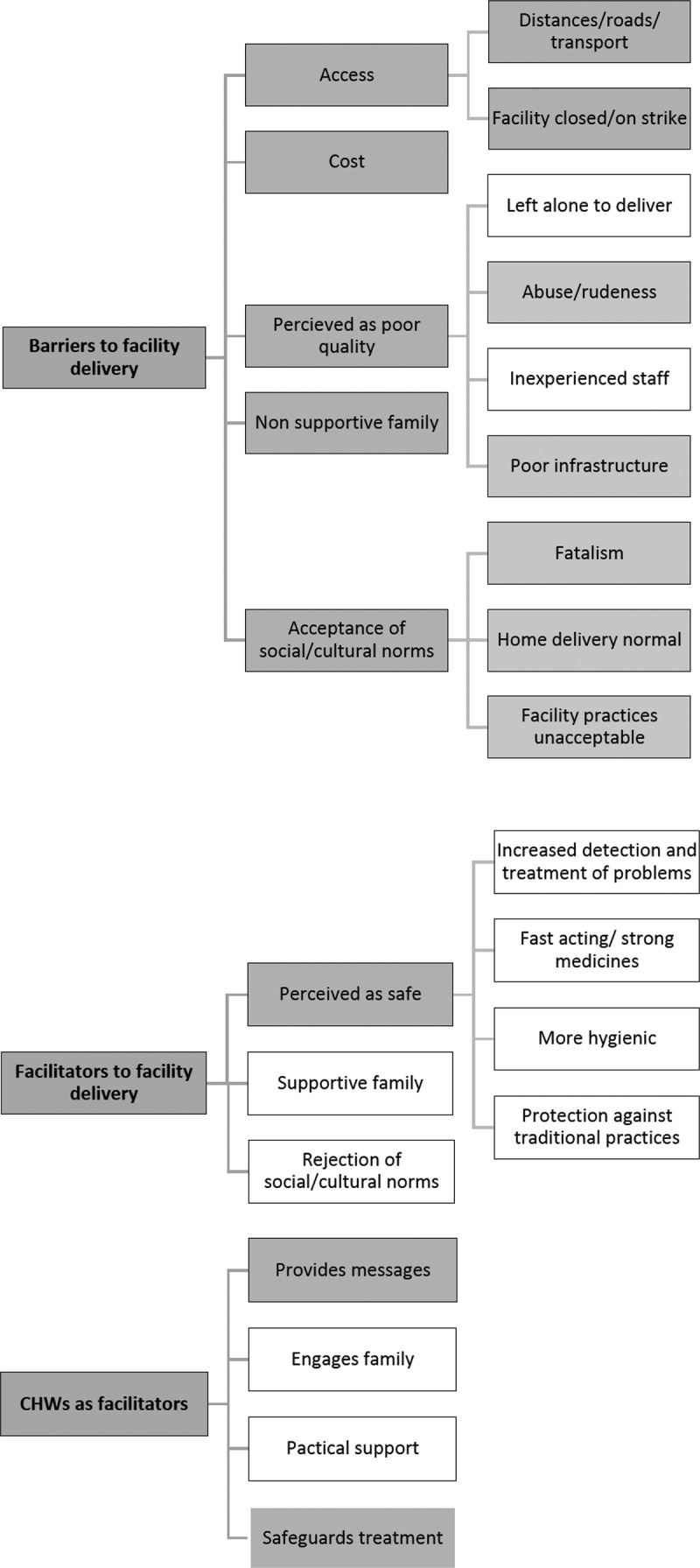
An overview of main results with themes, sub-themes and supporting codes (Grey = Deductive codes, White = Inductive codes).

The analysis resulted in three major themes with 12 supporting sub-themes. An overview of the results is given in Figure 1, which illustrates the coding frame and shows which themes are deductive (grey) and inductive (white) and also gives examples of inductive and deductive codes within the themes. The first of the three major themes is *‘barriers to a facility delivery’*, which consists of five sub-themes: *‘access’, ‘cost’*, that facilities are ‘*perceived as poor quality’*, a ‘*non supportive family’* in relation to a facility delivery, and *‘social/cultural norms’*. Under the sub-theme *‘access’* two further categories emerged: *‘distance, road and transport’* issues and the risk that the *‘facility is closed/on strike’*. The sub-theme around *‘perceived as poor quality’* has four categories: a perception that women will be *‘left alone to deliver’* in a facility, that they may face *‘abuse/rudeness’* at the facility, that the facility may have *‘inexperienced staff’* and *‘poor infrastructure’*. The sub-theme *‘social/cultural norms’* has three categories ‘*fatalistic beliefs’* that events were out of human control, beliefs that a *‘home delivery is normal’* and a perception that some ‘*facility practices are unacceptable’*, for example, birthing positions.

The second major theme is *‘facilitators to a facility delivery’* which consists of three sub-themes: that facility deliveries are ‘*perceived as safe’*, the *‘rejection of social/cultural norms’* with a desire to be modern, and that the woman has a *‘supportive family’* in relation to facility delivery. Under the sub-theme *‘perceived as safe’* four categories emerged: ‘*increased detection and treatment of problems’*, the availability of *‘fast acting and strong medicines’*, facilities being *‘more hygienic’* than home births and *‘protection against traditional practices’* that may be carried out during a home delivery and that the woman may not like or want.

The final major theme was *‘CHWs as facilitators’* of facility delivery with four sub-themes: CHWs *‘providing messages’* on the importance of facility delivery, that CHWs *‘engage with family members’ to* enlist their support for a facility delivery, provision of *‘practical support’* such as transport and CHWs *‘safeguarding treatment’* for example by reducing abuse  from health staff.

Below we present major themes and their sub themes. Social/cultural norms and family support were both facilitators and barriers to facility delivery and are thus presented together in a separate section. We did not see any variation in themes by the age or parity of the respondent, but our ability to compare and contrast data was limited as only 3 women had one child and only four were over 35 years of age.

## Barriers to facility delivery

### Sub-theme access

Long distances and road conditions were cited by all respondent groups as being a key barrier for facility delivery in more remote communities but not for the well-connected study sites: *‘Women in the villages live very far away from hospitals, they would like to go but they can’t’* [Grandmother FGD2] … … *‘Now that we are approaching rainy season, … … access road is a serious problem because all the routes are bad’* [CHW FGD3]. Transport issues were cited for more remote communities either because transport was unavailable, lacked fuel or because drivers refused to transport women in labour: *‘The owners of the vehicles they will tell you they don’t carry women in labour they are afraid the woman will deliver in their car or spoil their car’* [Mother FGD1] … …. *‘At times you look for vehicle you won’t find or there is no fuel …. In the midnight you won’t get motorcycle ….Seriously there is the problem of vehicle’* [CHW FGD4]

Lack of access to an open facility at night was also reported by all respondent groups: *‘In the night there is no one in the hospital …. Should we wait on the front steps till morning? In that case is it not better for the TBAs to come and deliver the babies at home?’* [Grandmother FGD1]. In the study sites women had a choice of facilities and if they had the resources could choose private care when public facilities were unavailable.

### Sub-theme cost

Cost was raised by all respondent groups, but was a particularly strong theme amongst fathers who were responsible for paying for transport and facility costs: *‘Most of the time it’s the money … if the husband knows that he cannot afford the hospital he will just pretend as if he does not agree …. the real truth is he has no money, let’s be honest with this’* [Father FGD1]. The very poor were perceived as not even considering a facility birth: *‘My husband said that, he doesn’t have even a penny … … We had no option than to leave her* [co wife] *at home and pray to God for intervention’* [Mother FGD3].

### Sub-theme facilities perceived as poor quality

All respondent groups reported perceived poor quality of care. Of particular importance to respondents was a perception that in some facilities women were not cared for and were left alone during delivery either because of understaffing or because health workers discriminated against them because of poverty or religion:

If you go to deliver in the health facility, the workers would leave you alone on the bed, when labour starts there would be no one to assist you. That is the reason why some women don’t want to deliver at the facility [Mother FGD3]

There is discrimination …. they would not attend to you …. The child may come out and they are not bothered …. they don’t care [Narrative mother 6]

This theme also included verbal and physical abuse by health workers some of whom were considered to be arrogant, unkind and prejudice: *‘They are just arrogant; when you tell them your problems, they will not listen to you. And when they talk to you, they raise their voice at you’* [IDI mother 2] … … *“She* [daughter] *said she was not going back again since they* [health workers] *drove her away … ‘they told me LEAVE AND GO BACK HOME as if I am a dog ….’* [CHW FGD1]. When husbands were unhappy with the treatment they sometimes decided to return home: *‘They tell them words that is not sweet …. Hurtful words ….he* [husband] *took her home … … because of the way they insulted the wife’* [Father FGD1]. In our study sites women had a choice of facilities and their choice was in part driven by perceptions of quality and in part by accessibility.

Facilities were also considered poor quality when they were overcrowded, unclean and with inexperienced health workers: *‘I delivered at home, the hospital near my house is dirty, I rather … we do our thing neatly at home … it’s better than delivering in that dirty place’* [Mother FGD1] …. *‘The ones* [health workers] *brought nowadays, they don’t know anything* [murmurs of agreement], *and they are arrogant’* [CHW FGD1].

## Facilitators to facility delivery

### Sub-theme facilities perceived as safe

The dominant themes for what motivates people to have a facility delivery, reported by all respondent groups, was that a facility delivery is safer for both the mother and baby, with a common sentiment being that: *‘nobody will know what to do’* if a problem occurs at home. Safety was related to the ability of staff to detect/manage problems early due to their training, the medicines available, and good hygiene:

I definitely trust the hospital staff more than the birth attendants ….they are better educated …. it is clear who has more knowledge [IDI mother 1]

Hospital delivery is better, your blood will be tested …. but at home you would not be able to get that …. you will just be doing things blindly [Narrative mother 3]

If it were to be at home, they will say they give smoke, bitter water or henna water ….but in the facility, they give them injection or drugs [Father FGD2]

Hospital is a place of delivery not someone’s house …. [at home] where the [delivery] mat is where you keep your slippers …. You see a child urinating where your wife is to deliver …. that place [hospital] it is a place that is neat and cleaned [Father FGD3]

Injections for bleeding and drips for long labour were frequently mentioned as efficacious medicines. The quick acting *‘hospital’* medicines were contrasted with bitter tasting herbal treatments given at home with facility delivery protecting women from home birth practices that they did not like: *‘As soon as they give you an injection you feel better, pills as soon as you take them you feel better …. when they give herbs, you take them for days and days and only when you get lucky you feel better’* [Narrative mother 2].

The safety of the facility was particularly salient for women who had experienced difficulties in the current pregnancy or in previous deliveries: *‘why would you stay and risk your life. Especially in my own case, with all the troubles I faced in the beginning, the best thing for me was the hospital’* [Narrative mother 2].

## Facilitators and barriers for facility delivery

### Sub-theme presence or absence of a supportive family

Fathers and grandmothers were key decision-makers in relation to delivery location: *‘The opinion of my husband, that is my opinion’* [Narrative mother 1], and they both positively and negatively influenced facility delivery. Fathers acted as facilitators for those who used facilities *‘He* [husband] *is the one who will look for the ride himself and say let’s go’* [Mother FGD2]. They were perceived as having the final say in decision-making and wielded financial control: *‘He did not stop her* [mother] *from going; he just refused to give her money’* [Mother FGD1]. Overall grandmothers were reported as having old-fashioned beliefs about home delivery that can be difficult to *‘withstand’* because of the power dynamics within households, but for those who delivered in facilities they were generally supportive: *‘they* [grandmothers] *are now telling us that we are very lucky, we now have better ways of doing things’* [Mother FGD1], and as having diminishing power: *‘it is their* [mothers and fathers] *time …. we have been swept into the dustbin’* [Grandmother FGD1].

Linked to the theme of decision-making was a sub-theme that a facility delivery protected women from the influence of their elders: ‘*You give birth at home … they will say you should go and bathe …. with leaves* …. *if you go* [to facility] *they* [health workers] *will set rules, in the eyes of your elders … and they will shield you a little … from the rules of the elders …. it protects you’* [Narrative mother 4].

### Sub-theme acceptance or rejection of social/cultural norms

Social norms around home births were reported by all respondent groups. These were related to fatalistic beliefs that events were out of human control: *‘He* [husband] *said no* [to a facility delivery] *that we should rely on God … he said if it is not her time nothing will happen to her …. he will come with a holy book’* [Grandmother FGD4] and beliefs that home births are normal births and the tradition: *‘Some don’t want to go because their tradition is to deliver at home … they are following the traditions of their parents’* [Mother FGD1]. This belief was felt to be particularly strong if a woman had previous safe deliveries at home ‘*She* [her friend] *feels that there is no use … she gave birth to all her children in her room without complication. No need’* [Mother FGD1].

There were also facility practices that were reported to be inconsistent with social norms, these included male attendants, insertions into the vagina and delivery position: *‘What stops me from delivery at the hospital is nothing but that lying down position of delivery ….if you go to the hospital, it is compulsory’* [CHW FGD1].

Those who used facilities had rejected these social norms, which they felt reflected women being *‘unenlightened’*, in favour of a belief in modernity: *‘We go with what is modern … … …. I am happy to be part of* the *people that are doing modern things …. I do not want to stay at home and deliver, that era has passed’* [Narrative mother 2] *… … ‘Modern day delivery, only in hospital*’ [Mother FGD4].

### CHWs as facilitators for facility births

CHWs were reported to have influenced facility delivery by providing messages on the benefits of a facility delivery, engaging with families, for example by convincing fathers of its importance, and appealing to mother’s desires to be modern: *‘I will give her* [mother] *an explanation that makes sense, and I will let her know that her mother’s era has passed. There is the need for you to know that when the music changes the dance step too has to change’* [CHW FGD2]. They were also reported to provide practical supports such as arranging transport: *‘Later when the labour pain increased, she* [CHW] *went out to look for a bike and when she got it, it took us to the hospital’* [Narrative mother 5].

A major and unanticipated sub-theme was the role that CHWs played in safeguarding better quality treatment. Several women reported that they had been accompanied by the CHW to the hospital for delivery and that, unlike family members, CHWs were sometimes allowed to be present during delivery: *‘We are like security. When the community women want to go to the hospital, they are afraid, but if they will go with them they have a cover and bad treatment will not be aimed at them …. the hospital staff shout at them but if we accompany them then they find some ease’* [CHW FGD1]. The presence of CHWs during the delivery provided women with social and practical support: *‘It was just me and her* [CHW] *alone in the delivery room … even when I want some water it is her that gives me …. she stayed with me up until the time I delivered’* [Narrative mother 4].

## Discussion

Our findings that decision-making power, accessibility, cost, social norms, perception of quality, and a fear of disrespect and abuse are barriers to facility delivery mirror the findings of other qualitative studies in Nigeria [10–14], and from low-income settings in general [9]. These findings highlight that interventions to increase facility delivery need to address a wide range of issues and work at multiple levels if they are to be successful [9].

The results presented above show that families valued facility deliveries as health workers had the abilities to detect and manage problems, and because of the quick acting and efficacious medicine available. Respondents contrasted the medicines available in facilities with the trial and error approach of using bad tasting herbs in home deliveries, and valued facility delivery as modern and civilized. We also found that previous negative birth experiences or a difficult pregnancy made the benefits more salient.

Our findings on disrespect and abuse during delivery care highlight a prevailing issue in many settings [36]. A study from Benue State found that experiencing disrespect and abuse did not impact intended use of facilities, as women still perceived facilities as the safest place to give birth and reported that practices such as health staff using abusive language and shouting as normative and expected [37]. In our study, we found that expectations and experiences of being left alone and uncared for during labour and delivery were reported as a particular barrier for facility delivery. This form of disrespectful care could have a more direct impact on perceptions of facilities as a safe place to give birth compared to the verbal abuse reported in Benue State.

While the barriers and facilitators we identified to facility delivery are not new, with systematic reviews reporting that barriers and facilitators to facility delivery are broadly similar across different country contexts [9,38], the role that CHWs can play in addressing these barriers has, to the best of our knowledge, not been previously studied. Of particular interest is the role the CHWs played in brokering better care in the facilities and their role as a source of support. Having a constant companion during delivery is recommended by WHO to improve satisfaction with services and labour outcomes [39]. Evidence from Kenya and Ghana suggests that women value having a companion during labour, mostly to provide instrumental support, but put less value on having a companion during the delivery itself because they feel that the companion cannot do anything, and they are embarrassed or fear gossip [40,41]. The need to better understand different forms of birth companionship has been acknowledged [40]. As was found in India [42], our study suggests that a more empowered companion may be beneficial in increasing respectful care.

The study has many strengths as it covers an important topic in an understudied geography with high maternal mortality. We utilized several qualitative methods in order to explore lived experiences; community perceptions, attitudes and beliefs, and social norms. We included a variety of respondents in order to elicit views from both women and family members who influence them. We were also able to triangulate our findings across data collection methods and respondent groups. Most previous research has focused on those who do not deliver in facilities whereas our study provides data on the drivers of positive behaviours as well as utilizing the fact that people may be better at explaining the behaviour of others than their own. However, more interviews with families who delivered at home and who are from more remote areas would have been desirable and might have resulted in more in-depth and nuanced findings, but the insurgency prevented this. Thus, the findings may not be transferrable to other areas with different contextual issues, especially those that are more remote and inaccessible or lack functioning CHWs. The small number of respondents who had one child and were over 35 years of age is also a limitation. The study relied on respondents reporting on their behaviours and indirectly on the behaviour of others. For the self reports there is the potential for social desirability bias and recall error, and for the indirect reporting there is the possibility of misrepresenting the behaviour of others. We tried to reduce social desirability bias and recall error by having short recall periods (3 months for narrative interviews), by asking respondents to reflect on the behaviour of others and by the use of methods in the FGDs that encouraged interaction and openness [34]. Triangulation was also used to help improve data validity. Despite these limitations, we feel that the key themes identified in this study have relevance to settings with similar cultural norms, accessibility issues and where perceptions of the quality of care are low.

### Conclusion

This study highlights both the facilitators and barriers to facility delivery, demonstrating the need for interventions that address a wide range of issues at multiple levels. We identify several areas in need of extra research and consideration, including the need to understand the differential impacts of different types of respectful care on facility utilization, and the role of family versus more empowered birth companions.

## Paper context

Qualitative studies in low-income settings identified factors such as decision-making power, accessibility, cost, social norms, perception of quality, and a fear of disrespect and abuse are barriers to facility delivery. We found similar barriers in North Nigeria, confirming the need for interventions that act at multiple levels. Our findings also suggest that some forms of disrespect and abuse may be greater barriers to facility delivery than others.
